# Ectopic Pleomorphic Adenoma: A Case Study and Scoping Review of the Literature

**DOI:** 10.7759/cureus.102785

**Published:** 2026-02-01

**Authors:** John Caraway, Alex Yang, Sophia Mckenzie, Noah Meltzer, Michael Noller

**Affiliations:** 1 Otolaryngology-Head and Neck Surgery, Walter Reed National Military Medical Center, Bethesda, USA; 2 Otolaryngology-Head and Neck Surgery, Uniformed Services University of the Health Sciences, Bethesda, USA; 3 Otolaryngology-Head and Neck Surgery, Kaiser Permanente, Rockville, USA

**Keywords:** carcinoma ex pleomorphic adenoma, carcinoma ex-pleomorphic adenoma, ectopic, heterotopic, pleomorphic adenoma, salivary gland

## Abstract

We present a case of an ectopic pleomorphic adenoma occurring in the glabellar region of an adult patient and perform the first known scoping review of the literature on ectopic pleomorphic adenomas of the head and neck, seeking to summarize their symptomatology, presentation, and treatment. Two independent reviewers performed searches and qualitative synthesis of relevant literature using PubMed, Embase, and Ovid All EBM Review databases. The authors adhered to the Preferred Reporting Items for Systematic Reviews and Meta-Analyses-Scoping Review (PRISMA-ScR) statement. Included studies reported ectopic pleomorphic adenoma originating above the clavicle. Exclusion criteria included tumors as a result of metastasis or of non-salivary gland origin. Forty-six case series and case reports of ectopic pleomorphic adenomas met the inclusion and exclusion criteria, for a total of 48 tumors. Most cases occurred during the 2nd to 6th decades of life with a male:female ratio of 1:1.2. Ectopic pleomorphic adenomas typically presented as a slow-growing, firm, mobile mass on a superficial region of the head or neck. Pain was reported in 19% of cases. All cases were treated with surgical excision, and there was only one recurrence after a malignant transformation.

## Introduction

Pleomorphic adenomas, also known as benign mixed tumors, are the most common benign neoplasm of the salivary gland, accounting for 84% of all benign salivary gland neoplasms [1,2]. They typically present as slow-growing, painless, solid, well-circumscribed masses with no facial nerve involvement [1,3,4]. While these tumors were in the past known as “mixed salivary gland tumors,” they were renamed “pleomorphic,” reflecting their morphologic diversity, as they are thought to originate from an uncommitted cell with both epithelial and mesenchymal tissue potential [1,3]. In 1972, the World Health Organization (WHO) published its first classification system for salivary gland neoplasms and subdivided adenomas by pleomorphic or monomorphic, but several lesions classified as monomorphic were neither truly histologically monomorphic nor monocellular, creating obvious confusion [5]. Thus, in 1991, an update to the histological classification for salivary gland tumors distinguished other types of benign salivary gland lesions, such as myoepitheliomas, basal cell adenomas, and Warthin tumors [5,6].

The main treatment paradigm for these tumors involves surgical excision with either parotidectomy or extracapsular dissection due to the risk of malignant transformation, which increases from 1.5% to 10% from 5 to 15 years of diagnosis, respectively [1,2,4,7]. A rapid increase in size, pain, facial nerve dysfunction, and fixation to adjacent structures are all indications of possible malignant transformation [1,4]. Pleomorphic adenomas also histopathologically possess satellite nodules or pseudopod-like extensions of the tumor, predisposing them to recurrence if micrographic portions of the tumor are left behind [1,4]. Radiotherapy use is limited, although it has been reported in a handful of cases of unresectable margins, multifocal recurrences, or poor surgical candidates [1,4].

Pleomorphic adenomas can occur in any salivary tissue, but they primarily occur in the parotid gland (85%), followed by the minor salivary glands (10%) and the submandibular glands (5%) [1,8]. Though in rare cases, pleomorphic adenomas have been known to occur in ectopic salivary tissue [9,10]. Willis et al. proposed three primary hypotheses of how ectopic salivary gland tissue develops: abnormal differentiation of the local tissues (i.e., heteroplasia), atypical persistence and development of vestigial structures, or dislocation of a rudimentary organ during the migration of developing tissue, the latter of which remains the leading theory [11]. Ectopic salivary tissue most commonly occurs throughout the head and neck region; however, it has been reported in several other anatomical areas below the clavicle, uncharacteristic of the normal pathway of salivary gland development [9].

In this scoping review of the literature, we seek to consolidate the topic of pleomorphic adenomas occurring in ectopic salivary gland tissue and comprehensively review their symptomatology, presentation, and treatment. Additionally, we also present our own case of an ectopic pleomorphic adenoma occurring in the glabellar region, which is notable given that no known embryologic salivary gland structures exist in this area [12].

## Case presentation

**Figure 1:**
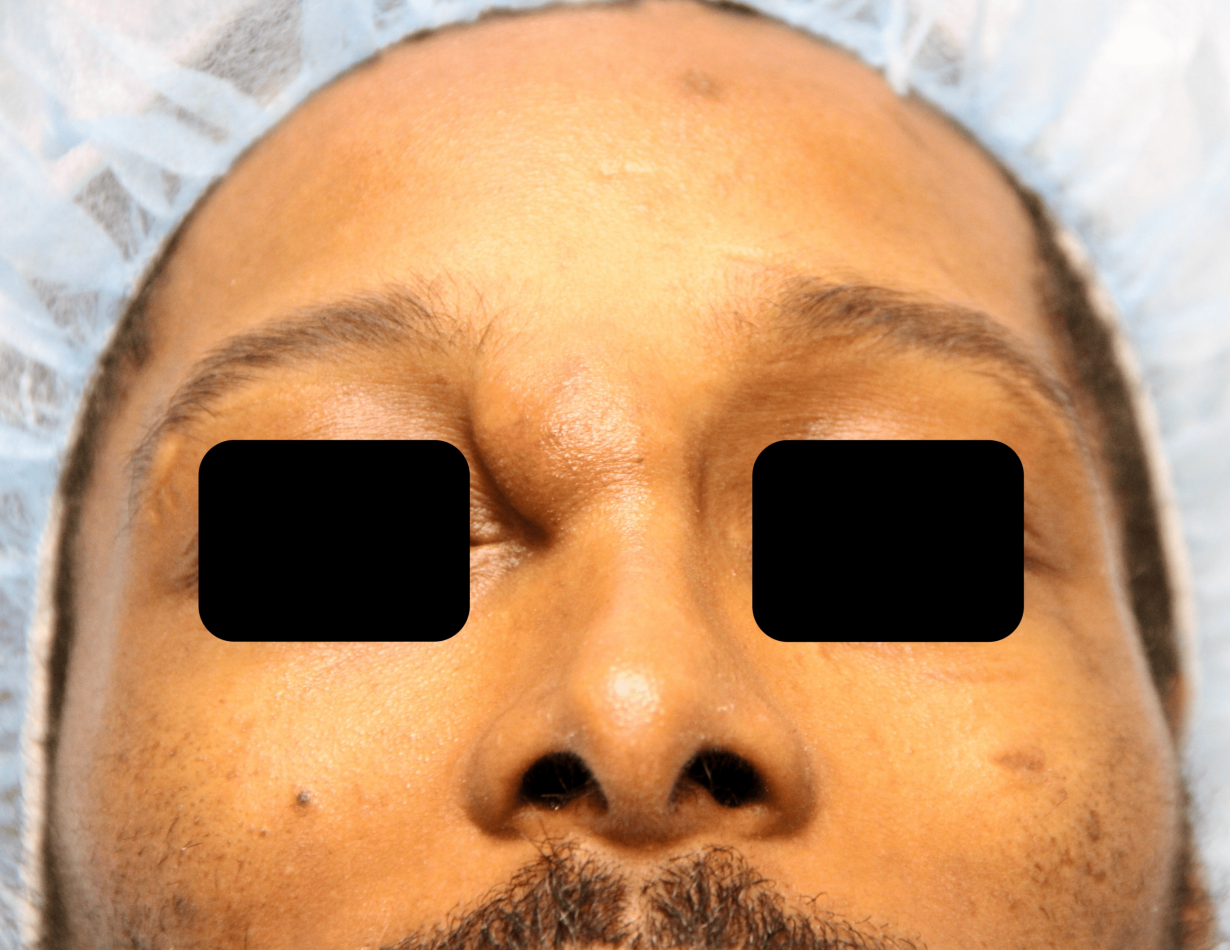
Preoperative frontal view Preoperative image (frontal view) of the patient with an arrow pointing to a large mass within the glabellar region

**Figure 2:**
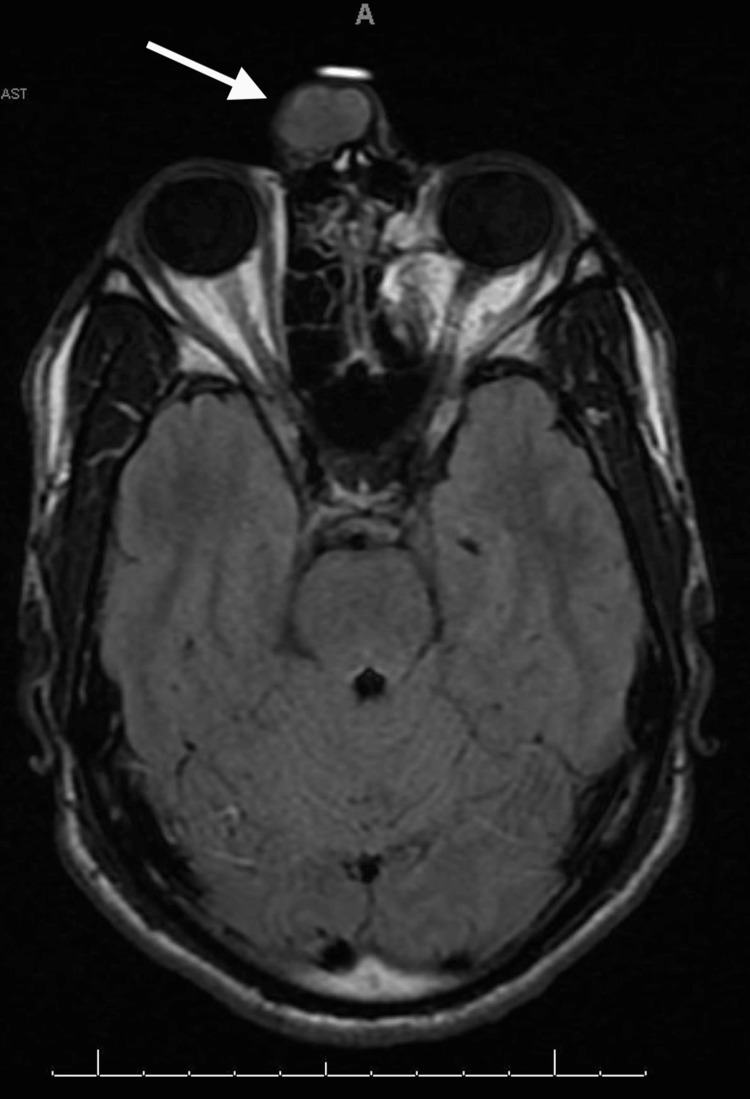
Preoperative MRI (pre-contrast T1-weighted) Preoperative MRI: axial cut of a pre-contrast T1-weighted image with an arrow demonstrating a hypointense mass within the glabellar region MRI: Magnetic resonance imaging

**Figure 3:**
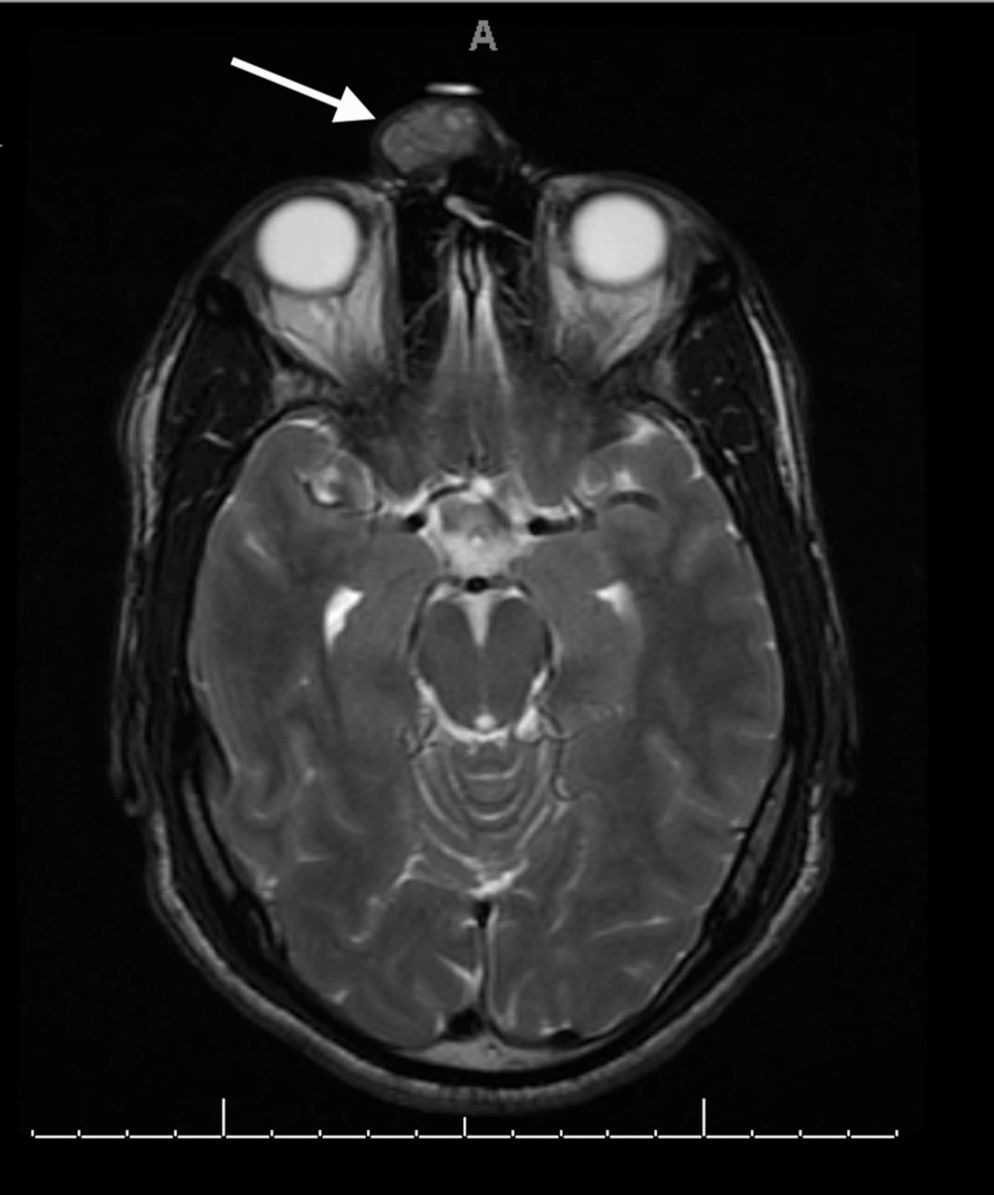
Preoperative MRI (pre-contrast T2-weighted) Preoperative MRI: axial cut of a pre-contrast T2-weighted image with an arrow demonstrating an isointense mass within the glabellar region

**Figure 4:**
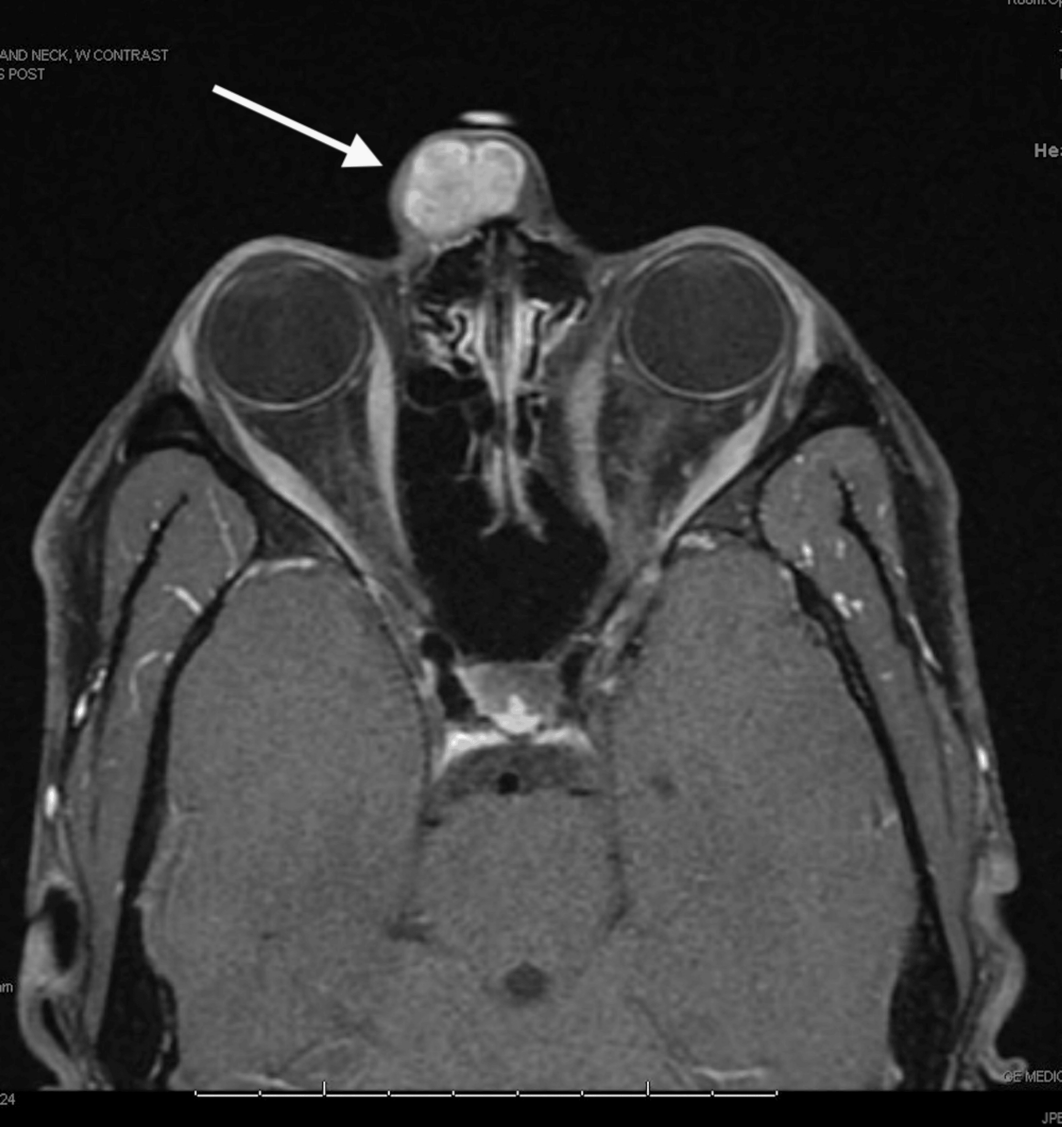
Preoperative MRI (post-contrast T1-weighted) Preoperative MRI: axial cut of a post-contrast T1-weighted image with an arrow demonstrating a contrast-enhancing mass within the glabellar region MRI: Magnetic resonance imaging

**Figure 5:**
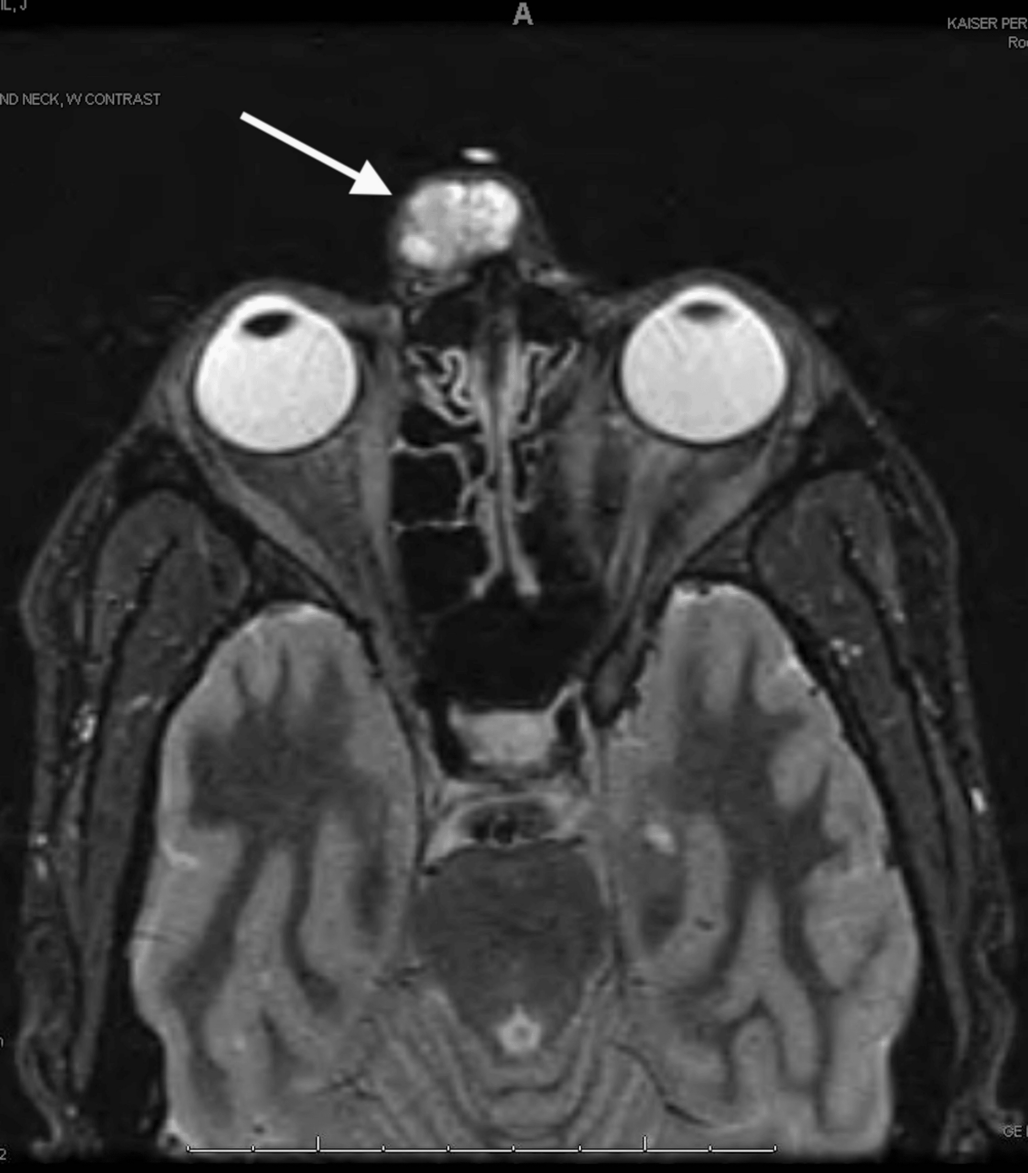
Preoperative MRI (post-contrast T2-weighted) Preoperative MRI: axial cut of a post-contrast T2-weighted image with an arrow demonstrating a contrast-enhancing mass within the glabellar region MRI: Magnetic resonance imaging

**Figure 6:**
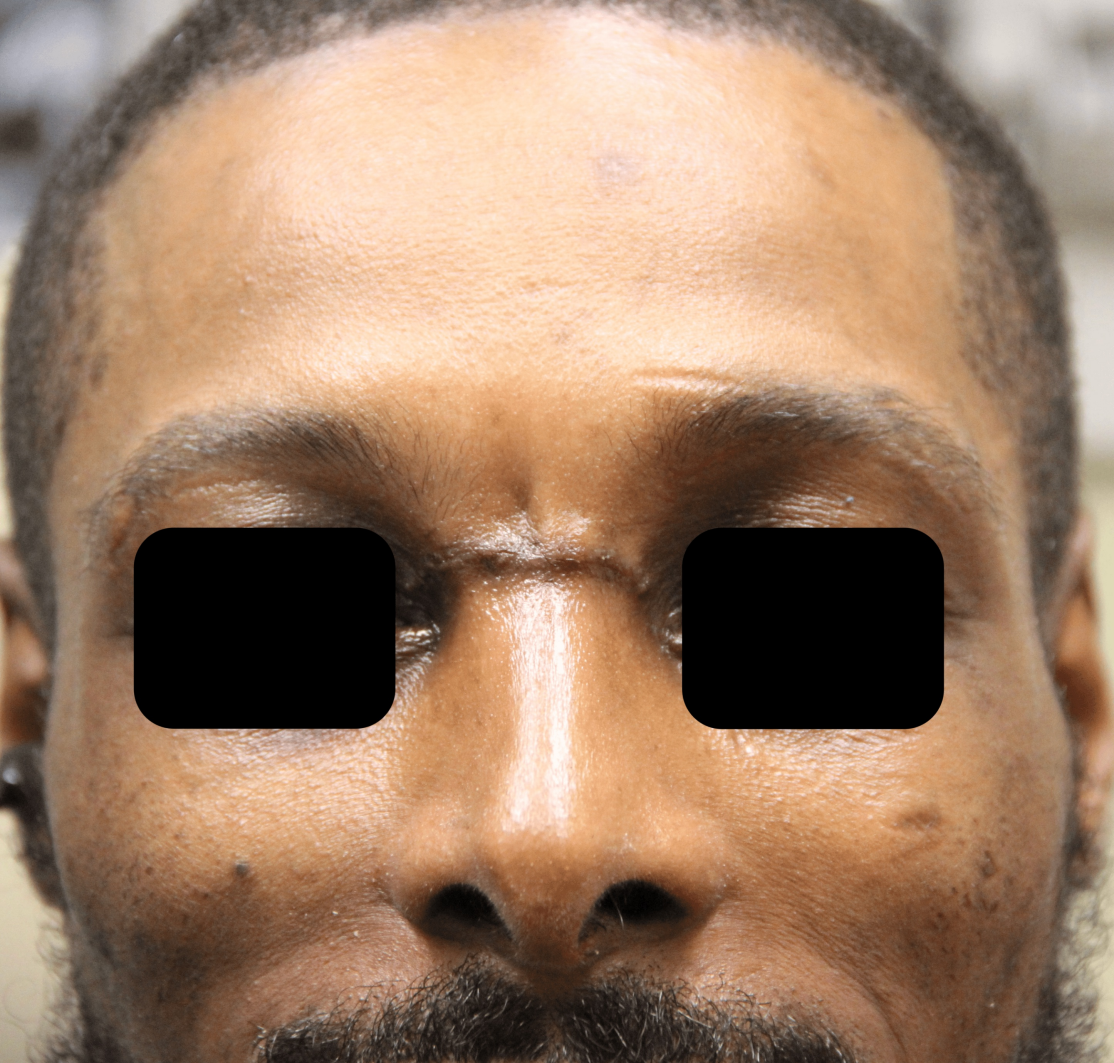
Postoperative frontal view Postoperative image (frontal view) of the patient with an arrow demonstrating a well-healed incision where the glabellar mass had been excised

The patient was a 31-year-old male referred to the facial plastic and reconstructive surgery clinic for a slowly enlarging, non-tender right nasal canthal facial mass. The mass was first appreciated in 2009, shortly after experiencing blunt trauma to the region. It progressively enlarged and affected his ability to wear glasses. Examination revealed a glabellar, 2 cm, mobile, soft tissue mass in close adherence to the skin (Figure 1). Multiplanar, multisequence MRI with contrast of the face revealed a superficial 2.1 x 1.6 x 2.0 cm heterogeneous T2 isointense, T1 hypointense, enhancing mass abutting the right medial canthal segment (Figures 2-5). In-office fine needle aspiration was performed and revealed mostly single plasmacytoid epithelial cells present in a background of a myxoid metachromatic fibrillary matrix; scattered ductal-like epithelia were also observed. Immunohistochemistry showed strong reactivity to keratin stains, CK7, AE1/AE3, vimentin, and S100, and light immunoreactivity with p53. There was no reactivity to CD34, HMB-45, Mart-1, and GCDFP15. A preliminary diagnosis of pleomorphic adenoma was made, and after appropriate counseling, the patient opted to proceed with surgical excision. The procedure was performed under general anesthesia. Blunt dissection down to the supraperiosteal plane just deep to the mass was performed through a small superiorly based skin incision. The mass was freely mobilized from the surrounding soft tissue, resected en bloc, and sent for histopathological examination. Postoperative recovery was uneventful, and the patient was very pleased with his postoperative appearance (Figure 6). Final histopathological examination confirmed the diagnosis of pleomorphic adenoma. The final maximum tumor dimension was 2.4 cm. At one year of follow-up, the patient had no evidence of recurrence.

## Discussion

Methods

A scoping review of the literature was performed using the Preferred Reporting Items for Systematic Reviews and Meta-Analysis-Scoping Review (PRISMA-ScR) strategy [13]. A PCC (population, concept, context) statement was observed to assess studies for inclusion and exclusion. PICOS criteria are as follows: a) patients: all patients with ectopic pleomorphic adenomas, b) concept: to synthesize the presentation, symptomatology, disease course, and treatment modalities of ectopic PAs compared to traditional PAs, and c) context: International reports and studies evaluating ectopic pleomorphic adenoma.

Information Sources and Search Strategy

A systematic literature search was performed of Embase, PubMed, and Ovid All EBM Reviews (HTA, Cochrane, ACP Journal Club, DSR, CCTR, CMR, DARE, and NHSEED) from May 4, 2022, to June 1, 2024, with filters for English and articles after 1991. Medical Subject Heading (MeSH) terms included pleomorphic adenoma, ectopic, heterotopic, facial bones, head, neck, parathyroid glands, thyroid gland, auricular, cutaneous distal, ear canal, intracranial, intraosseous, lip, lips, lymph node, mastoid, mandible, mandibular, maxilla, middle ear, ocular, orbital, nasal, nose, parapharyngeal, parathyroid, periocular, postauricular, postseptal, preauricular, preseptal, preseptal, scalp, sinus, subcutaneous, supra-clavicular, supraclavicular, and thyroid. Various combinations of MeSH terms were used with AND/OR statements to refine results.

Study Selection and Data Collection

**Figure 7:**
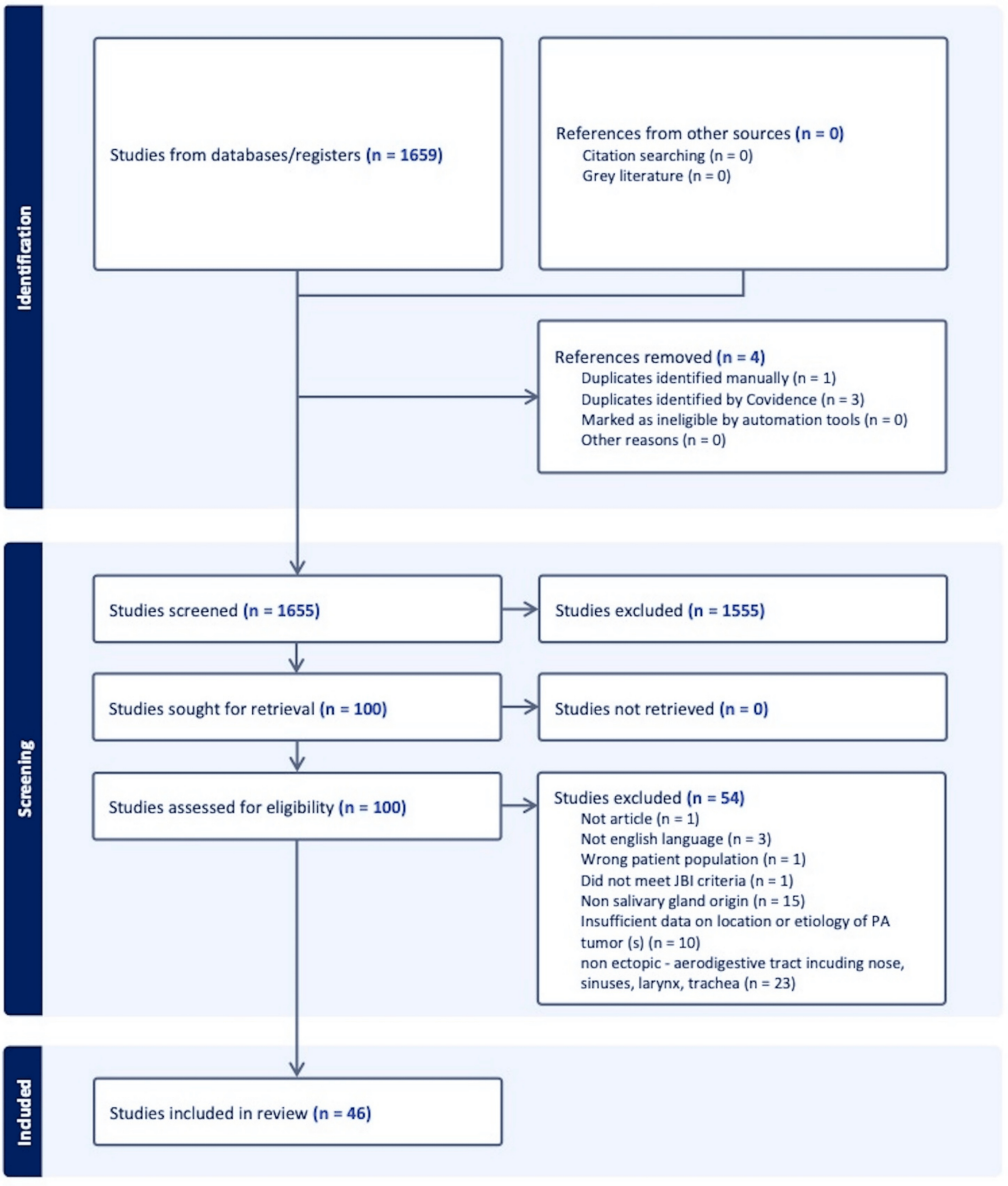
PRISMA flow diagram

Included studies were written in English, reported ectopic pleomorphic adenomas originating above the sternal notch, and occurred after 1991, corresponding to the updated WHO histological classification update [5]. Studies were excluded if tumors originated from non-salivary sources (i.e., sebaceous gland or lacrimal gland); non-ectopic pleomorphic adenomas arose from minor salivary gland tissue within the mucosa of the aerodigestive tract (i.e., nasal cavity, nasopharynx, paranasal sinuses, hard or soft palate, lips, buccal, oral cavity, pharynx, and trachea); metastasis after malignant transformation; non-pleomorphic adenomas; or chondroid syringomas. Two reviewers (JC, AY) first screened titles and abstracts and then downloaded appropriate full-text versions, independently reviewing each article for inclusion. A third author (MN) resolved conflicts. A PRISMA flow diagram of study selection is displayed in Figure 7. Data was extracted by two independent reviewers (AY, JC) and verified by a third reviewer (SM). Data extracted from each study included demographics, location, size, largest dimension, imaging modality, management, histopathology, recurrence time (if any), pain, growth rate, mobility, and firmness.

Quality Assessment

A critical appraisal of included studies was conducted to ensure that all sources of evidence used within the scoping review were reliable and without bias. Quality assessments of downloaded articles were reviewed with the Joanna Briggs Institute (JBI) appraisal checklist for the quality of case reports [14]. Two reviewers (JD, AY) scored each case report, and their scores were then averaged. Any case report with an average score of less than 6 (out of 8) was excluded. 

Results

Forty-six case series and case reports of ectopic pleomorphic adenoma were included, for a total of 48 tumors, of which 43 were benign while five had undergone malignant transformation (Table 1) [15-60].

**Table 1:** Case report/series demographics and tumor characteristics *The Joanna Briggs Institute Critical Appraisal Tool for Case Reports Used in Systematic Reviews: 1. Were the patient’s demographic characteristics clearly described? 2. Was the patient’s history clearly described and presented as a timeline? 3. Was the current clinical condition of the patient on presentation clearly described? 4. Were diagnostic tests or methods and the results clearly described? 5. Was the intervention(s) or treatment procedure(s) clearly described? 6. Was the post-intervention clinical condition clearly described? 7. Were adverse events (harms) or unanticipated events identified and described? 8. Does the case report provide takeaway lessons? Each component is rated either 0 or 1 for a total score out of 8. A score >=6 was considered adequate for inclusion within our scoping review.

First Author	Year	JBI*	Age	Gender	Location	Imaging	Histopathology	Diameter (mm)
Aghaghazvini [15]	2015	6	38	M	Left mandibular parasymphysis	Dental X-ray	pleomorphic adenoma	15
Alsaleh [16]	2020	8	20	F	Left infratemporal fossa	CT	pleomorphic adenoma	40
Arora [17]	2015	7	40	F	Right tragus	None	pleomorphic adenoma	20
Arunkumar [18]	2011	6	8	M	Right neck	None	pleomorphic adenoma	35
Asai [19]	1995	6	1	F	Right mandibular angle	Dental X-Ray	malignant myoepithelioma ex pleomorphic adenoma	20
Ashraf [20]	2007	6	40	F	Neck	None	pleomorphic adenoma	40
Aver [21]	2002	7	31	F	Left maxilla	Dental X-ray	pleomorphic adenoma	20
Badia [22]	1996	7	41	F	Right nasal bone	CT	pleomorphic adenoma	8
Baldi [23]	2003	6	40	M	Right neck	None	pleomorphic adenoma	50
Chimelli [58]	2000	7	44	M	Suprasellar region	MRI	pleomorphic adenoma	25
Dasegowda [24]	2018	7	15	M	Right nasal dorsum	None	pleomorphic adenoma	20
El-Hadi [25]	2009	7	59	F	Right infratemporal fossa	CT	pleomorphic adenoma	45
Evans [26]	1991	6	11	F	Left mandibular angle	None	pleomorphic adenoma	25
Evans [26]	1991	6	15	M	Right mandibular angle	US	pleomorphic adenoma	30
Gelidan [27]	2021	6	46	M	Left philtrum column	None	pleomorphic adenoma	10
Grome [28]	2016	7	38	F	Left postauricular region	None	pleomorphic adenoma	15
Hakeem [29]	2009	7	20	M	Left parapharyngeal space	CT	pleomorphic adenoma	80
Hakeem [29]	2009	7	53	M	Right parapharyngeal space	CT	pleomorphic adenoma	60
Hallak [30]	2020	7	72	M	Left supraclavicular region	CT	pleomorphic adenoma	30
Hsu [31]	2018	6	32	F	Right postauricular region	CT	pleomorphic adenoma	25
Ismi [32]	2015	7	72	F	Right submandibular region	MRI	carcinoma ex pleomorphic adenoma	75
Jeyanthi [33]	2007	7	45	F	Right infratemporal fossa	CT	pleomorphic adenoma	60
Kamath [34]	2015	6	39	M	Right submandibular region	US	pleomorphic adenoma	50
Kanazawa [35]	2000	7	52	F	Left pterygopalatine fossa	CT, MRI	pleomorphic adenoma	31
Kesse [36]	2002	6	65	F	Right parapharyngeal space	MRI	pleomorphic adenoma	Not Stated
Kim [37]	2019	7	43	F	Left nasolabial fold	None	pleomorphic adenoma	7
Kumar [38]	2011	7	28	M	Left auricle	None	pleomorphic adenoma	25
LaMaccia [39]	2017	7	55	F	Left neck	CT	pleomorphic adenoma	35
Levy [40]	2012	7	66	M	Right thyroid isthmus	US	pleomorphic adenoma	15
Luksic [41]	2012	7	34	M	Right submandibular region	CT	pleomorphic adenoma	23
Mahafza [42]	1999	7	50	F	Right neck	No	pleomorphic adenoma	35
Mouzali [43]	2019	7	20	M	Right ala nasi	None	pleomorphic adenoma	20
Ojha [44]	2007	7	71	F	Right mandibular body	Dental X-ray	pleomorphic adenoma	10
Panigrahi [45]	2013	7	16	M	Left masticator space	CT, MRI	pleomorphic adenoma	38
Poondiyar Sirajuddin [46]	2021	7	34	M	Left infratemporal fossa	CT, MRI	pleomorphic adenoma	50
Ramasamy [47]	2021	7	67	F	Right supraclavicular region	CT	carcinoma ex pleomorphic adenoma	39
Ray [48]	2016	7	54	F	Left parapharyngeal space	CT	pleomorphic adenoma	60
Rodgers [49]	1991	6	14	M	Right neck	None	pleomorphic adenoma	20
Surana [50]	1993	6	12	F	Right neck	None	pleomorphic adenoma	30
Takahashi [51]	2011	7	56	M	Suprasellar region	CT, MRI	pleomorphic adenoma	Not Stated
Tanrivermis [52]	2017	6	40	F	Right infratemporal fossa	CT, MRI	carcinoma ex pleomorphic adenoma	40
Tay [53]	1995	6	50	M	Left neck	None	pleomorphic adenoma	18
Varghese [54]	2003	7	40	M	Right parapharyngeal space	CT	pleomorphic adenoma	60
Vegari [55]	2012	6	21	F	Right neck	CT	pleomorphic adenoma	48
Yang [56]	2016	8	71	F	Left posterior fossa	CT, MRI	pleomorphic adenoma	47
Yano [57]	1997	8	35	F	Left posterior fossa	CT, MRI	pleomorphic adenoma	63
Zhang [59]	2015	7	23	M	Suprasellar region	CT, MRI	pleomorphic adenoma	Not Stated
Zhao [60]	2011		13	F	Mandibular body	Dental X-ray	carcinoma ex pleomorphic adenoma	300

Demographics

The mean age of occurrence was 39, and the median age of occurrence was 40. Age distribution was diverse, ranging from a one-year-old female to a 72-year-old male. 81% of cases occurred during the 2nd to 6th decades of life. The male-to-female ratio was 1:1.2. There was no difference in the distribution of age based on gender. The majority of reported cases occurred in Asia (60%), followed by Europe (17%), North America (13%), Africa (4%), South America (4%), and Australia (2%).

Presentation and Symptomatology

In 42/48 cases (88%), ectopic pleomorphic adenomas presented as a mass growing in an unexpected region of the head/neck. Five cases (10%), all of which were intracranial, presented with symptoms of headache and/or changes in vision that prompted further workup. In only one case was an ectopic pleomorphic adenoma found incidentally on imaging [40]. They occurred in numerous locations, with 14 cases (29%) in superficial facial regions, seven cases (15%) in deep facial regions, 14 cases (29%) in the superficial neck, eight cases (17%) in the deep tissue of the neck, and five cases (10%) within the cranium. In the majority of cases (46/48, 96%), ectopic pleomorphic adenomas presented as a slow-growing mass noticed years prior. Rapid growth was only described in two cases, both of which involved malignant transformation of the tumor [19,52]. Pain was a reported symptom in 9/48 cases (19%); however, only one of these involved a malignant tumor. These were located in the posterior fossa, infratemporal fossa, mandible, and parapharyngeal space and could be as small as 2 cm [19].

*Tumor Characteristics* 

Most ectopic pleomorphic adenomas were described as mobile (37/48, 77%) and firm (47/48, 97%). While 11/48 (23%) of cases reported that the lesions were “non-mobile,” the majority of these were still benign. Size varied dramatically between lesions, with the largest dimension ranging from 0.7 cm to 30 cm. The average largest dimension was 3.4 cm (excluding one outlier measuring 30 cm), with 50% of these being discovered with a largest dimension < 3 cm. There were 5/48 cases (10%) in which malignant transformation was confirmed on pathologic examination. These consisted of one case of malignant myoepithelioma in a pleomorphic adenoma and four cases of carcinoma ex pleomorphic adenoma [19,32,47,52,60]. Regarding the features of these malignant cases, 40% were non-mobile, 40% reported rapid growth, 20% reported pain, and they all occurred in different regions of the head/neck.

Management and Recurrence

Initial workup included a variety of imaging, such as dental X-ray, computed tomography (CT), or magnetic resonance imaging (MRI). Specifically, CT imaging was utilized in 20/48 (42%) of cases, and MRI was used in 11/48 (23%) of cases. MRI was always employed in cases involving an intracranial lesion. In all cases, treatment was with complete surgical excision. No study reported the use of radiotherapy or chemotherapy. Reported follow-up durations were inconsistently reported across studies, ranging from 6 to 36 months. Only one case of an ectopic tumor reported recurrence within a year of follow-up, in which the tumor had gone through malignant transformation [19].

Discussion

While there are numerous reports in the literature of ectopic pleomorphic adenomas, the case presented in this manuscript is unique in that it is the first known case of ectopic pleomorphic adenoma occurring in the glabellar region, where no embryologic salivary gland tissue is known to exist [12].

Our review finds that ectopic pleomorphic adenomas occur in a similar population to that of traditional pleomorphic adenomas. Traditional pleomorphic adenomas most often occur during the 4th-6th decades of life and are more common in females [3,4]. O’Brien conducted a study involving 254 patients with pleomorphic adenoma and reported a median age of 46 with a male-to-female ratio of 1:1.7 [61]. Similarly, in a study by Laccourreye et al. with 229 patients, a 1:1.4 male-to-female ratio was reported [62]. Ectopic pleomorphic adenomas appear to have similar findings, with 81% occurring between the 2nd and 6th decades of life and a similar male-to-female ratio of 1:1.2.

Benign salivary gland tumors, such as pleomorphic adenomas, often present as a slow-growing, solid, mobile, painless mass in the region of a major or minor salivary gland; however, they are also commonly found incidentally on imaging [1,3,4]. In contrast, most ectopic pleomorphic adenomas presented as a mass in an unexpected area of the head or neck, five cases of which were located intracranially and presented with symptoms of headache and/or vision changes. Like their non-ectopic counterparts, ectopic pleomorphic adenomas were mostly slow growing (96% of cases), other than two cases of rapid growth consistent with a malignant transformation [1,19,52]. Additionally, ectopic pleomorphic adenomas presented similarly in size to non-ectopic tumors. A retrospective study conducted by Da Silva et al. of 1086 patients with pleomorphic adenomas reported the mean size at the time of diagnosis was 3 cm, while the mean size for ectopic tumors in our study was 3.4 cm Notably, though, in 30% of ectopic cases, tumors were less than 2 cm at the time of diagnosis, which is smaller than is typical for non-ectopic variants [64]. The superficial locations for many of these ectopic tumors likely resulted in them being discovered at a relatively smaller size. Finally, nine ectopic cases (19%) presented with pain, most of which were interestingly benign. Pain is often an omen of malignant transformation in a traditional pleomorphic adenoma [1,4], whereas in ectopic variants, there may be some earlier involvement of subcutaneous nervous fibers or early symptoms due to compression. In our review, symptoms of pain did not appear to correlate with size, and lesions as small as 1 cm presented with pain [44].

Management of ectopic neoplasms was identical to traditional pleomorphic adenomas, with surgical excision being the preferred treatment [1,3,4]. In our study, no ectopic cases reported employing postoperative radiotherapy, which in some instances has been used in the treatment of pleomorphic adenomas (i.e., cases with positive margins, multifocal recurrences, or poor surgical candidates) [1,4]. Recurrence of pleomorphic adenoma is rare, with control rates reported as 99% or higher [1,4,61,62]. Similarly, only one ectopic case reported recurrence, and it involved the transformation of the ectopic pleomorphic adenoma into a malignant myoepithelioma tumor [19].

Our review identified several controversies regarding the definition of ectopic salivary gland tumor, which proved to be a limitation in this study. There are numerous minor salivary glands located throughout the aerodigestive tract, including the oral cavity, nasal cavities, oropharynx, pharynx, larynx, and trachea [1,10]. Thus, it can be difficult to determine whether a pleomorphic adenoma is arising from ectopic salivary tissue or simply a physiologic minor salivary gland. We attempted to define ectopic pleomorphic adenoma as any pleomorphic adenoma occurring in salivary tissue outside these normal locations of the aerodigestive tract, but this proved challenging. For example, pleomorphic adenomas arising from the deep lobe of the parotid gland can present within the parapharyngeal space [1]; however, we identified multiple cases in which pleomorphic adenomas occurred in what was reported as de novo salivary gland tissue within the parapharyngeal space lateral to the pharyngeal constrictor muscles. It was hypothesized that this de novo salivary tissue was likely derived from displaced or aberrant salivary gland tissue within a lymph node rather than normal minor salivary gland tissue, which is thought to exist within the aerodigestive mucosa medial to the pharyngeal constrictor muscles [29,48,54]. Additionally, pleomorphic adenomas of a similar appearance can arise from lacrimal glands, sebaceous glands, or sweat glands [1,65-67]. Chondroid syringomas, initially termed benign mixed skin tumors of the salivary type, arise from the sebaceous and sweat glands of the skin [67,68]. They most often present as a slow-growing, firm, mobile, painless mass located on the nose, cheek, upper lip, scalp, forehead, and chin. Although they are generally found in the head/neck region, they can localize to any region of the body. They are often mistaken for epidermal cysts, given their superficial location with no attachment to deep tissue [68]. Chondroid syringomas resemble pleomorphic adenomas histologically and cytologically, with similar immunohistochemical markers such as keratin stains, vimentin, and S100 [67]. Therefore, they are typically distinguished based on the location of occurrence or the presence of normal salivary gland tissue within the same histopathological specimen; however, this cannot always be done with certainty [69,70]. To our present knowledge, there is no accurate way to differentiate chondroid syringomas from ectopic pleomorphic adenomas. We reported a case of pleomorphic adenoma occurring in the glabellar region that we believe derived from ectopic salivary tissue, as its location was likely too deep for sebaceous or sweat glands to exist. Additionally, ductal-like elements more consistent with a salivary gland tumor were found within the final surgical histopathological specimen; however, like other cases, there was no documented method of determining with certainty whether this lesion was an ectopic pleomorphic adenoma or a chondroid syringoma. Cases describing chondroid syringomas were ultimately excluded from this review, though it may be possible that these two tumor types are the same entity but of different morphologies. Further research is needed to better characterize the two tumor types, though both are rare occurrences in the head and neck and may be difficult to study.

Other limitations to this review include the use of only case reports or case series, which increases the risk of bias due to the subjective nature of such studies. However, given its rarity, it is not plausible to perform retrospective reviews; it appears most surgeons only encounter such a phenomenon a few times in their careers. Nevertheless, our study’s aim is to summarize the current state of knowledge regarding ectopic pleomorphic adenomas.

## Conclusions

Ectopic pleomorphic adenomas can occur throughout the head and neck region and should be on the differential for a head or neck mass. They typically present as a slow-growing, firm, mobile mass with a propensity for superficial regions of the head/neck and, in some cases, can be painful. We hypothesize this may be related to their atypical location of the head or neck. Malignancy was only present in 10% of cases. In almost all cases, surgical excision was an effective treatment, and recurrence was extremely rare.
